# Death, treatment decisions and the permanent vegetative state: evidence from families and experts

**DOI:** 10.1007/s11019-013-9540-y

**Published:** 2014-01-19

**Authors:** Stephen Holland, Celia Kitzinger, Jenny Kitzinger

**Affiliations:** 1 Department of Philosophy, University of York, York, YO10 5DD UK; 2 Department of Sociology, University of York, York, UK; 3 School of Journalism, Media and Cultural Studies, Cardiff University, Cardiff, UK

**Keywords:** Defining death, End-of-life, Euthanasia, Nutrition and hydration

## Abstract

Some brain injured patients are left in a permanent vegetative state, i.e., they have irreversibly lost their capacity for consciousness but retained some autonomic physiological functions, such as breathing unaided. Having discussed the controversial nature of the permanent vegetative state as a diagnostic category, we turn to the question of the patients’ ontological status. Are the permanently vegetative alive, dead, or in some other state? We present empirical data from interviews with relatives of patients, and with experts, to support the view that the ontological state of permanently vegetative patients is unclear: such patients are neither straightforwardly alive nor simply dead. Having defended this view from counter-arguments we turn to the practical question as to how these patients ought to be treated. Some relatives and experts believe it is right for patients to be shifted from their currently unclear ontological state to that of being straightforwardly dead, but many are concerned or even horrified by the only legally sanctioned method guaranteed to achieve this, namely withdrawal of clinically assisted nutrition and hydration. A way of addressing this distress would be to allow active euthanasia for these patients. This is highly controversial; but we argue that standard objections to allowing active euthanasia for this particular class of permanently vegetative patients are weakened by these patients’ distinctive ontological status.

## Introduction

How shall we regard those in [PermVS]? They are periodically awake, and their bodies breathe and digest on their own. These traits bespeak life. Yet they are not conscious and never will be: subjectively, this is death (Wikler 1988, p. 41)

Catastrophic brain injuries have various causes including trauma due to accidents, anoxia (lack of oxygen) due, for example, to cardiac arrest, and illnesses such as viral encephalitis. Brain injured patients fall into various diagnostic categories. The vegetative state (VS) refers to patients who have suffered damage to parts of the brain responsible for consciousness but who retain sufficient brain stem activity to maintain some autonomic physiological functions, including spontaneous breathing and stable circulation.^1^ The minimally conscious state (MCS) was introduced as a diagnostic category in 2002 for patients who demonstrate minimal but clearly discernible behavioural evidence of awareness of themselves or their environment (Giacino et al. 2002).^2^ Patients in MCS show inconsistent, but reproducible, responses above the level of spontaneous or reflexive behaviour, which indicate some degree of interaction with their surroundings.

Of these diagnoses, this article focuses on the vegetative state (VS).^3^ This is further subdivided into ‘persistent’ (or ‘continuing’) VS on the one hand, and ‘permanent’ VS on the other. The subdivision is based on duration of the state of unconsciousness (in the absence of complicating but reversible factors which might suppress consciousness). In the UK, a patient who has been in VS due to anoxic or other metabolic injury for at least 6 months, or in VS due to trauma for 12 months, is diagnosed as being in a permanent VS.^4^ To diagnose a patient as being permanently vegetative is to predict that their loss of capacity for consciousness is irreversible.^5^ *Permanent* VS (PermVS) is the specific focus of this article.

We are acutely aware of the controversial nature of the permanent vegetative state. Specifically, ethical discussions such as ours are based on two premises: that PermVS exists, and that we can know which brain injured patients are in this state. Both premises are said to be belied by clinical realities. In particular, recent evidence suggests that some PermVS patients may retain a degree of conscious awareness; there are well publicised cases of patients emerging from what was thought to be a permanent vegetative state; and our current understanding of the neurological basis of consciousness is not sufficiently refined to diagnose a vegetative state as permanent with complete confidence (Fins 2008). Given this, it is suggested that PermVS is a hypothetical scenario—a thought experiment rather than a clinical reality—so philosophical discussion of the ethics of the treatment of the permanently vegetative is at best academic and at worst dangerous (Borthwick 1995).

We acknowledge that it is important to continue to clarify the clinical realities of the permanent vegetative state. But the implications of recent scientific work in this area are contested,^6^ and, whilst recent developments and refinements might enable the detection and correction of *misdiagnoses*, they do not establish that all vegetative patients retain a degree of conscious awareness.^7^ Regarding the epistemological problem, knowledge does not require certainty, so the premise that one cannot be certain that a patient is in a permanent vegetative state does not entail that one cannot know that they are. This is in keeping with the fact that medicine is rife with uncertainty; PermVS, like other diagnostic categories, admits of the possibility of error.^8^ Finally, diagnostic categories for brain injured patients may well be vague in the philosophical sense that there is no bright line between them; but vague boundaries are still boundaries so it remains plausible that a subcategory of patients are, and can be known to be, in PermVS.

Two other considerations suggest that discussing the ethics of treatment of PermVS patients is appropriate and urgent, not redundant. First, it is a medical reality that patients are currently diagnosed as permanently vegetative, and managed accordingly, so whether their treatment is ethical is a pertinent question notwithstanding ongoing investigation into the condition. Second, even if were true that all vegetative patients retain or regain conscious awareness, in many cases this would be extremely minimal and nothing akin to what is usually meant by a centre of consciousness. Discussion of the ethical treatment of this subcategory of brain injured patients is important even if they are not ‘permanently vegetative’ in the standard sense. In sum, discussions of the ethics of treatment of patients diagnosed as permanently vegetative should proceed notwithstanding the controversial nature of, and ongoing neurological research into, the condition.

A final introductory point concerns methodology. This paper engages with a bioethical issue by drawing on empirical research. The relationship between ethics and empirical research is vexed. The controversy centres on empirical ethics, ‘a broad category, grasping different interpretations of combining or trying to integrate ethics and empirical research’ (Borry et al. 2004, p. 1). A standard objection to empirical ethics is that it commits the naturalistic fallacy by deriving normative, evaluative, ethical conclusions from naturalistic—typically, social scientific—premises. In other words, empirical ethicists tell us what ought to be done on the basis of what people think, say, believe or do. There are various theoretical responses to this challenge (de Vries and Gordijn 2009) but our approach avoids the naturalistic fallacy altogether. This is because we do not base (bio)ethical conclusions (about how PermVS patients ought to be treated) directly on empirical data (relatives’ and others’ views). Rather, we use empirical data directly to support claims about the concept and nature of death and, specifically, the ontological state of PermVS patients. In turn, we draw bioethical implications from these conclusions. That the ontological status of a patient has normative significance is beyond doubt—for example, that a patient is dead entails that it can be permissible to retrieve their organs—so there is no dubious dialectical move in our argument from naturalistic premises to ethical conclusions.^9^

## The ontological status of PermVS patients

The general problematic is how permanently vegetative patients ought to be treated. There are various considerations, such as autonomy (respecting the wishes of the patient) and societal implications of end-of-life policies. The one we focus on here is the ontological status of the patient. That this is important is clear; for example, if they are already dead then harvesting transplant organs from PermVS patients who had wished to donate would not contravene the ‘dead donor rule’ that no one should be killed by organ retrieval. What, then, is the ontological condition of the PermVS patient: alive, dead, or some other state?

This turns on a debate about death centring on three related questions. (1) The definition of death: what is death? (2) The determination of death: what has to happen to a creature for it to die? (3) The diagnosis of death: how are we to test for the occurrence of death in particular cases? The current ethical and legal landscape is dominated by a biological paradigm: (1) death is irreversible breakdown in the functioning of the organism as a whole; (2) for human beings, this occurs when the brain is irreversibly incapable of maintaining integrative organismic functioning; (3) clinical tests for whether a brain is in this state have been devised, such as the apnea test (in some countries, such as the UK, death is determined by the state of the brain stem because irreversible loss of all brain stem functions is inevitably followed by holistic organismic breakdown). According to the biological paradigm, PermVS patients are alive because they display autonomic physiological functioning, despite their irreversible loss of consciousness.^10^

But the biological paradigm is contested. Notably, advocates of a consciousness-based paradigm for death claim: (1) death for human beings is irreversible loss of the capacity for consciousness; (2) this occurs when parts of the brain responsible for consciousness are irreversibly damaged; and (3) the principal diagnostic tests for death are techniques to establish, for example, a patient’s lack of awareness of themselves or their environment, and lack of response to stimuli. According to this consciousness-based paradigm, PermVS patients are dead despite autonomic physiological functioning because of their irreversible loss of capacity for consciousness.^11^

Holland (2010) has argued that paradigms such as the biological and consciousness-based are reductivist and fail to capture the complexity of the phenomenon.^12^ He suggests that clarifying the way death is ordinarily conceived is more important to defining death than alternative approaches, such as empirical investigation, metaphysical theorising, or asking experts. Our ordinary concept of death includes the definition familiar from the biological paradigm—i.e., death is irreversible breakdown in the functioning of the organism as a whole—but also involves thoughts such as, for someone who has died, it will never again be like anything to be them. So, our ordinary concept of death has at least two conceptual components, one biological (death is about how organisms cease to function), the other consciousness-based (death is about never again being a centre of consciousness). No doubt the concept of death is even richer, including perspectives provided by, for example, religious frameworks and idiosyncratic beliefs.

On Holland’s account, when faced with what are sometimes called ‘ambiguous cases’ such as PermVS patients, we are unsure about their ontological status. In other words, we struggle to understand whether they are alive, dead, or in some other state. On the one hand, these patients are alive according to the biological definition, which is central to our ordinary understanding of death. On the other hand, non-biological components of our ordinary concept—notably, the consciousness-based thought that death is a matter of never again enjoying any thoughts, sensations or experiences—fit the condition of a PermVS patient, which leads us to think and talk of them as being dead (or, at least, not straightforwardly alive).

## Data from interviews with relatives of PermVS patients

That the ordinary understanding of death includes, but is richer than, the biological definition, and that people struggle conceptually over the ontological status of PermVS patients, are claims about how real people think and talk, how they conceptualise matters. Are these claims true? We pursue this question by reference to empirical data from interviews with relatives of severely brain injured patients, conducted by the second and third authors, who themselves have a severely brain injured sister. Over fifty interviews have so far been completed. Although the focus of this paper is PermVS patients, other diagnostic categories mentioned above are represented in the study, including patients whose vegetative state is persistent but not yet permanent, minimally conscious patients, and some cases of uncertain diagnosis (for example, patients whose condition is borderline between vegetative and minimally conscious).

Interviews were semi-structured; an interview schedule was used but conversations were allowed to develop naturally in unforeseen ways. Interviews were recorded, transcribed and thematically coded. Extracts quoted here have been anonymised, and names of people and places are pseudonyms. Here we focus primarily on family interviews with respondents who have accepted a PermVS diagnosis for their relative (i.e., they report believing that their relative has lost, and is extremely unlikely ever to regain, awareness of self or environment). We also draw on a second data set compiled from interviews with professionals working on disorders of consciousness—such as consultant neurologists and legal experts—using a similar protocol (except that some respondents asked to be named; of these, only one is quoted here, James Howe, and all other names of family members and professionals used in this article are pseudonyms).^13^

Do the interview data reveal an understanding of death so rich and complex as to cause research participants to struggle with the ontological status of PermVS patients, in accordance with Holland’s analysis?^14^ Explicitly and implicitly, interviewees repeatedly speak of patients as being alive and yet to die. Such discourse clearly concords with the biological paradigm in which death is defined as irreversible breakdown in functioning of the organism as a whole: in that paradigm, PermVS patients are still alive because they maintain integrated autonomic physiological functions. So, for example, Tania, the mother of a PermVS son (pseudonymised as ‘Charlie’), states explicitly that he is still alive despite the fact that people she had thought of as friends no longer ask about him:

Tania: You know they’ll cross the road rather than speak to you and they talk about- Often they talk to me and they say “oh, how is Spencer [another son]?” or “how is your mum?” Very rarely will they ask about Charlie and I’m thinking, “He’s still alive!”

Another interviewee, Brian, implies that his relative is alive by way of contrast with his projected future death: “Yeah. While I’d be heartbroken if he died, it’s a funny- it’s like almost a- as if it would be a sense of relief if it was to happen.” Comments of this kind were so frequent as to suggest that this is a natural and familiar way for respondents to think and talk about the patients.^15^

But the crucial finding is that this does not fully capture how interviewees understand the patients’ ontological state. Specifically, at various points in interviews, respondents with a relative in a permanent vegetative state struggled to explain the patient’s ontological status by refusing to speak of them as straightforwardly alive, and even explicitly talking about them as being already dead. The first representative extract presented here is drawn from a joint interview with the patient’s brother, Harry, and Harry’s partner, Natalie.

Natalie: What we are convinced about is that uhm, from everything that we can find out, it is not in Zoe’s [the patient’s] best interest to be still alive. ‘Cause she’s existing. She isn’t living
Int: Mm
Harry: I mean there’s a line where it says clinicians are good at fixing bodies but they’re not good at fixing brains
Int: Yeah. You’ve said she’s existing, not living, and I think you [Harry] said earlier she- that the Zoe you knew died 4 years ago
Harry: She did, yeah
Int: Uhm, how do you make sense of the body in the bed? Is she- it’s kind of between life and death somehow?
Harry: No she- As far as I’m concerned- Well yeah, obviously, it’s between life and death. You’re in no man’s land, basically.

The interviewer’s phrase ‘the body in the bed’ is resonant with the experience of many family members whose relatives are in PermVS, and is used spontaneously by some interviewees, along with references to the patient as a ‘shell’ or a ‘husk’. For example, Jade comments,

It feels like it’s just a body. It’s Colin’s body being kept alive somehow. He’s not in it anymore […] It’s just a shell. It’s a shell of a body. It’s so- so damaged, the brain. I feel that it’s not Colin anymore. […] It isn’t a life. Is it even an existence?

Likewise, Rhiannon says, “We don’t want to lose them. We want to keep them here with us. But all you’re keeping is a shell.”

Brian also describes his brother’s body as a “shell”, and uses a range of other formulations to try to capture his brother’s current ontological condition: “the body’s there but the engine’s gone”; “there’s a case there and somebody’s taken the motherboard out”; and, “as the old saying goes, the lights are on but there’s nobody in”. Although Brian was quoted earlier as saying that he’d be “heartbroken if he [his brother] died”—thereby implying that his brother is not dead—elsewhere in the interview he also talks about his brother as *already* dead: “I don’t mean this nastily or anything else like that- but possibly to me, Aaron died the day [of the assault that led to his brain injury]”. At several points in the interview he states in quick succession both that his brother is “already dead” and that he is “not dead”; for example,

Brian: He’s already dead. The only reason he’s not dead is because his heart pumps […] And we’re not sure whether he reasons because we don’t know enough. But what we do know, or what information we have got at this present time, is he’s effectively dead

This struggle to articulate what being ‘effectively dead’ amounts to emerges when Aaron is compared to friends who are ‘really dead’:

Brian: I said, “So Aaron hasn’t got a life to lead. Or live.” And I suppose that is the difference between Aaron and my friends that have died, right? They’ve died. Their life’s ended, and it’s gone. Aaron is alive but he hasn’t got a life to live. I don’t know if that makes sense.^16^

Another interviewee, whose mother had died after being vegetative for more than 3 years, displays similar uncertainty, on the one hand agreeing that his mother effectively died in the car crash that caused the brain injuries leading her to become permanently vegetative (such that he “didn’t believe she was really there anymore”) but also talking about how he treated her body “just in case I was wrong”:^17^

Int: From your point of view, did you lose your mother, did she in effect die in the accident?
Tim: Yeah. Yeah. Of course it’s more comforting to think of it like that. So I suppose that that’s what I latched onto
Int: So how did you relate to the body in the bed, that was –
Tim: Oh well, not in a- not- (laughs) Yeah, you might think that you’d just be kind of careless or uh, or dismissive of it, but not at all. […] On the one hand I was confident and comfort- I was comfortable with the idea of withdrawing nutrition because I didn’t believe that she was- uh was really there anymore. And if she had been there she would have hated it. But on the other hand, just in case I was wrong, I would- I and everybody else involved would be- would treat her with dignity and respect and try and look after her.^18^

The same uncertainty about ontological status arises in interviews with professionals working in the area of disorders of consciousness, such as court expert witnesses, including consultant neurologists. For example, we interviewed the neurologist, James Howe, who was involved with the ground-breaking case of Tony Bland which established that treatment withdrawal could be legally permitted for PermVS patients in the UK.^19^ In this interview we pressed Howe on Bland’s ontological status at the point of treatment withdrawal:

Int: And when you withdrew treatment from Tony Bland, you didn’t feel you were killing him? You felt he’d been killed by the Hillsborough disaster?
Jim: He was already dead. Mr Bland [his father] said that he was already dead. “My son was already dead.”
Int: But his heart was beating. He was breathing unaided.
Jim: Yes, that’s right. Yeah.
Int: How is that dead?
Jim: Well, it is dead because what matters is consciousness. […] With the extinction of consciousness then the individual is dead. It doesn’t matter what your heart’s doing; it’s just a pump.
  […]
Int: So for you, not being dead means being conscious, at least some of the time?
Jim: Yes. Yes, that’s right.^20^

Howe’s comment are so forthright that he might seem to be claiming that Tony Bland is straightforwardly dead; but he advocated treatment withdrawal precisely in order to shift Tony Bland’s ontological status to that of ‘really dead’.

In sum, evidence from interview data accumulates to support the view that our ordinary concept of death is more complex than that of a solely biological phenomenon, and this creates conceptual uncertainty about the ontological status of PermVS patients.^21^

## Literal and metaphorical uses of ontological concepts

One objection to the foregoing is that when interviewees say the PermVS patient is alive and not dead, they are using these terms literally; by contrast, when they talk of the patients being dead, they are speaking metaphorically: ‘Only living organisms can die … Use of the word ‘death’ or ‘die’ outside of this strict biological context is acceptable but is metaphorical’ (Bernat 1998, p. 15). Does empirical evidence support this objection? One reason for thinking not is based on a discernible contrast with discourse pertaining to minimally conscious patients. The MCS patient is alive on all standard definitions of death (and on Holland’s original analysis because, notwithstanding its complexity, our ordinary understanding of death does not extend to thinking of patients whose consciousness is very minimal as dead). But relatives of MCS patients sometimes seem to equivocate over their loved-one’s ontological status. Nonetheless, it seems clear that there is not the serious struggle to conceptualise MCS patients which characterises interviews with respondents who have a relative in PermVS. This next extract, from an interview with someone whose brother is in MCS, is illustrative.

Int: And is there a part of you that feels you’ve lost your brother?
Trevor: Yes. Yeah, there is ‘I’ve lost my brother’ but, you know, if we start wallowing in ‘lost your brother’, the only way to be positive is to help him to get back.
Int: Yep.
Trevor: If I, at the beginning, thought I’d lost my brother, blah, blah, blah, he wouldn’t be where he is now. The attitude is you’ve got to try and get him back, give him the chance. I’m sure if he didn’t want to live, he could easily have died. He could have easily died himself.

For this interviewee, the patient in MCS is hard to communicate with and changed from who he was, but clearly not dead. He takes a positive perspective on “help[ing] him to get back” to something like the life he had. Another interviewee whose relative was in MCS but has subsequently died of natural causes describes her interaction with him in a way that clearly displays that, in her view, not only was he definitely not dead, but also he was sufficiently alive to have the agency and communicative ability to convey to her that he wanted to be dead:

Elspeth: He appeared to lean forward and tell me that he wanted to die- in obviously not so many words […] And at another point as well where I said to him- he was in so much pain, breathing really difficult and I said “Ian, I just wish there was something I could do.” And he again leant out and looked at me. And to me that meant ‘there is something you can fucking do’… And it just—it suddenly became really clear that that’s what we had to do is to help him do that. And [that’s] when we went to see the lawyer [to get advice about withdrawing treatment].

For both Trevor and Elspeth their brother is clearly alive, though Trevor believes his MCS brother *wants* to remain alive, whereas Elspeth believes her MCS brother would rather be dead. The patient has ‘lost the life they had’ and each is (according to their sibling) reacting to that in a different way. So here the distinction between literal and metaphorical discourse about the patients’ ontology is apposite: respondents’ talk of the MCS patient being alive is literal; their acute sense that the person they knew before the injury having ‘gone’ leads them to speak metaphorically of their being dead. By contrast, the discourse illustrated in the previous section about PermVS patients is not metaphorical but, rather, expresses respondents’ struggle to articulate the PermVS patients’ ontological state.^22^

## Practical implications

What are the practical implications of the unclear ontological status of PermVS patients? We pursue the issue that is uppermost for the interviewees. All the interviewees who accepted the PermVS diagnosis at some point in their interview stated that they wanted the situation to be resolved by their relative being moved from their currently unclear state to having clearly died, even though such a shift might unleash a new layer of grief alongside a sense of relief.^23^ For example, Tania comments,

I will be brutally honest and say all I have wanted for a long time is for Charles to be at peace. People say to me, still, you know, “You must never give up hope; there’s always hope.” But after almost 9 years, I’m sorry but my hope is that Charles finds peace.

Far from being thoughtless, such views accord with established principles of medical ethics, notably that treatment should be in the best interest of the patient, and respect for patient autonomy. For example, Tim comments, “I couldn’t say that her best interests were served by maintaining the functionality of her body until she died of old age in 20 years’ time” and, “I know that she would have been greatly distressed if she could have known that she would have been condemned to live this long in such a condition.”^24^

Does the current legal situation support these views? PermVS patients are maintained by life-sustaining interventions, principally, artificial nutrition and hydration (ANH). Often (and from here in this article) this is referred to as ‘clinically assisted nutrition and hydration’ (CANH) to draw attention to its status as medical treatment. On the basis of precedents such as *Quinlan*, *Cruzan*, and *Schiavo* in the US, and *Bland* in the UK, it is legally permissible (indeed, even appropriate) under certain circumstances to apply for a court order to withdraw CANH.^25^ But many interviewees are horrified by the prospect of their relative being treated in this way. This is understandable notwithstanding the presumption—which, recall, is never certain—that patients diagnosed as permanently vegetative are incapable of experiencing unpleasant sensations.

Tania: We hated it. They reassured us that, you know, “Oh he would be sedated, he wouldn’t feel any pain.” But we would have to sit there for up to 3 weeks to, basically, watch him die. Craig [patient’s brother] said, “but it’s so awful Mum- I couldn’t bear for them to do that to Charles”. There’s no way I’d ask for it. No way.

Harry concurs: “There are ways and means of doing it compassionately, but instead we’re going to withdraw nutrition and hydration when we could actually […] give her a drug to go to sleep forever.”

Some of the neurologists we interviewed agreed that withdrawal of treatment was not in the best interests of the patient when there were quicker ways of bringing about their death. For example, one consultant neurologist recommended palliative sedation.

Neurologist: I think that the means of death needs to be as quick and easy and painless as both the law and the clinical team themselves are prepared to do. The law tells you at the moment that you cannot actually inject insulin or other agents […] then a decent amount of sedatives, not sort of injecting enough to kill them immediately, but to make sure that progressively and rapidly- so it’s under some sort of control
Int: So- okay. So palliative care that is-
Neurologist: Sort of what you might call positive palliative care rather than reactive palliative care
Int: Terminal sedation?
Neurologist: Yeah.

The same neurologist puts the point by reference to the doctrine of double effect (though this was not appealed to in *Bland*):

Neurologist: … once we say in the court or wherever we say we’re going to withdraw hydration, I mean, we’re essentially saying we’re going to kill this person. I mean, there is no other outcome. And we’re doing this knowing that’s going to be the outcome, there is no other benefit, there is no- It’s not a sort of second- you know, when you give morphine to relieve pain and you happen to think-
Int: Double effect
Neurologist: That’s right. There’s no double effect of this. There’s only a single effect. The withdrawal of hydration causes death. And if there is a double effect it’s distress, which is hardly in the person’s best interest. So, you know, we are quite sanguine I suppose about the fact we are killing them, but we’re doing it in a very slow and laborious and nasty way.^26^

Another consultant neurologist—reflecting on his response to a young adult PermVS patient who was the subject of a court case for withdrawal of CANH which was eventually approved—was even more forthright: “I used to sometimes stand and look at him and think, ‘if you were my son I would kill you right now with my own two hands’. I really felt that, because it’s just awful.”

Such views—explored more fully in Kitzinger and Kitzinger (2014)—raise the question as to whether other ways of dealing with these patients should be permitted, alongside withdrawing CANH. We argue that our data support the case for reconsidering active euthanasia specifically for PermVS patients as one of the options to be debated.^27^ Many relatives strongly believe that actively euthanizing PermVS patients is more in the latter’s interests than is the current method of withdrawing CANH; this in itself counts in favour of permitting this option. Furthermore, the case for considering active euthanasia for PermVS patients is strengthened by our analysis of their ontological status. In particular, that permanently vegetative patients are in an unclear ontological state weakens arguments against permitting active euthanasia for them. We have space here to provide two illustrative examples.^28^

A standard objection to permitting active euthanasia for any class of patients, including PermVS patients, is that it will create a slippery slope to objectionable killings. There are standard responses within the literature, such as the lack of evidence of a slippery slope from health care systems that permit forms of active euthanasia (Marquet et al. 2003). Our response is different. A *logical* slippery slope exists when the reasons for acting in one case also apply to another case; a *psychological* slippery slope exists when, even though two cases are logically distinct, agents have a psychological predisposition—no doubt grounded in socio-cultural institutions—to slide from one to the other. Our claim that PermVS and MCS are dissimilar in a distinctive way—namely, they are ontologically dissimilar—adds further confirmation that these patients/conditions are logically distinct: i.e., no logical slippery slope exists from PermVS to MCS so the reasons for acting in the one case do not apply to the other case.^29^ In turn, this weakens the claim that there is a psychological slippery slope from, say, permitting active euthanasia for PermVS patients to killing MCS patients.^30^

Another standard objection to active euthanasia is that the distinction between active and passive euthanasia maps onto the distinction between killing and letting die, killing is worse than letting die, so passive euthanasia is permissible, but active euthanasia is not. Again, there are familiar responses in the literature, such as that the killing/letting die distinction cannot be maintained (Brody 1992; cf. Kopelman 2007) and that killing and letting die are morally equivalent (Rachels 1975; Tooley 1980; cf. Nesbitt 1995; Hanser 1999). Again, our argument is different. The distinctions between active and passive euthanasia, and between killing and letting die, are less pertinent in the case of patients whose ontological status is unclear, than in cases of patients who are straightforwardly alive. This is because the point of the appeal to the killing/letting die distinction is to avoid agents actively ending innocent people’s lives. But a PermVS patient is not straightforwardly alive in the first place; in turn, it is unclear what moral work the killing/letting die distinction could do in this particular case. It is not providing a bulwark against killing innocent people because the PermVS patient is not a standard victim of a killing; rather, they are a patient in an unavoidably unclear ontological state who is not straightforwardly alive, and who currently can be legally treated in such a way as to ensure that their ontological status is that of being straightforwardly dead.

In sum, our analysis suggests that we debate the possibility of active euthanasia as a legally permitted option for PermVS patients. Of course, on the basis of considerations such as best interest and autonomy, active euthanasia might be declined in favour of other forms of treatment. For example, it might be decided that a patient ought to be maintained in their current vegetative state, or allowed to die by withdrawing CANH, on the strength of their previous religious convictions and other strongly held values and beliefs.^31^ Nonetheless, we are sympathetic to a ‘shift of burden’ advocated by some bioethicists: i.e., changing from the current default position often adopted of assuming that life-sustaining interventions will be continued, whilst allowing applications for withdrawal, to assuming life-sustaining interventions will be *discontinued* after a clearly specified period, whilst allowing applications for their continuation (Angell 1994; Constable 2012).

## Concluding remarks

Empirical data support the view presented in Holland (2010) that the ontological state of permanently vegetative patients is unclear: they are neither straightforwardly alive nor straightforwardly dead. Some relatives and experts take the view that the least worst option in this situation would be to shift patients from their currently unclear ontological state to that of being clearly dead. But many are concerned, or even horrified, by the prospect of the only legally sanctioned method guaranteed to achieve this, namely withdrawal of clinically assisted nutrition and hydration. Our analysis supports the case for debating a policy of allowing active euthanasia for PermVS patients (subject to all the sort of safeguards that are now or will be in the future put in place for allowing their deaths from treatment withdrawal). Views expressed in interviews provide a reason in favour of this legal and social policy change, which would be more acceptable to some families, less distressing for them, and more likely to allow them to go along with a ‘best interests’ decision which respects a patients’ prior expressed wishes. Additionally, objections to allowing active euthanasia—for example, based on putative slippery slopes or the killing/letting die distinction—are compromised by the distinctive ontological status of PermVS patients.

## Footnotes

1 Clinicians sometimes use ‘consciousness’ to refer to both wakefulness and awareness; patients in VS exhibit wakefulness, including eye closure and opening which give the appearance of a sleep-wake cycle, but lack awareness of themselves or their environment. In accordance with philosophical usage, however, in this article we restrict ‘consciousness’ to meaning conscious awareness.

2 Conditions related to—and sometimes confused with—MCS which are not the focus of this article include locked-in syndrome and long-term profound cognitive and physical disabilities.

3 We are aware that the term ‘vegetative state’ is not universally condoned—notably, in mainland Europe (see, e.g., Laureys et al. 2010)—but we prefer it because it remains well established in the UK where the interviews we draw on were conducted (Royal College of Physicians 2003).

4 Lengths of time are disparate because the likelihood of recovery from anoxic injury is lower than that of recovery from brain trauma; see Royal College of Physicians (2003, 14). Incidentally, another RCP Working Party report has gone to press as we write—the third author of the present article is a member of the committee—with the new guidelines being published in December 2013 (Royal College of Physicians 2013). In the USA, the Multi-Society Task Force on Persistent Vegetative State concluded that a patient’s condition is ‘permanent three months after non-traumatic and 12 months after traumatic injury’, where ‘permanent’ means that recovery is ‘exceedingly rare’ (Multi-Society Task Force 1994).

5 By contrast, ‘whole brain death’ (WBD) occurs when all parts of the brain – those responsible for consciousness and those responsible for physiological functions—are irreversibly damaged such that physiological activity, including breathing and circulation, requires mechanical support. The controversy over the fact that whole brain dead patients are declared legally dead in the UK and the USA is well documented (Miller and Truog 2010) and have recently received extensive media publicity with public debate about two US cases, Jahi McMath and Marlise Muñoz (e.g., http://www.nytimes.com/2014/01/10/health/the-science-behind-brain-death.html?hp&_r=3). See also the neurologist quoted in footnote 19.

6 Monti et al. (2010). See also Cruse et al. (2011), Goldfine et al. (2013), Dyer (2013). For a response to diagnostic uncertainties see Fischer and Truog (2013).

7 Hence, ‘results show that a small proportion of patients in a vegetative or minimally conscious state have brain activation reflecting some awareness and cognition. Careful clinical examination will result in reclassification of the state of consciousness in some of these patients’ (Monti et al. 2010, 579).

8 Though the rate of error in diagnosing and differentiating cases of PermVS and MCS is notably high; see Andrews et al. (1996), Schnakers et al. (2009).

9 For sustained discussion of empirical ethics, see Vol 5, no. 1 (2004) of this journal; Vol 23, no. 4 (2009) of *Bioethics*.

10 For presentation, defence, refinement, and application of the biological paradigm, see, respectively, Bernat et al. (1981), Bernat (1998, 1999), Lamb (1996).

11 An early advocate of consciousness-based accounts is Veatch (1975). Lizza (1993, 2006) has consistently defended higher brain accounts, including arguing that a creature goes out of existence with the loss of essential properties; for human beings, personhood is an essential property; consciousness is necessary for personhood; so the PermVS patient, who has irreversibly lost capacity for consciousness, is dead because they have lost an essential property. See also Rich (1997).

12 Holland distances this account from similar sounding views, such as the ‘two-deaths’ view that human beings literally die two deaths (that of the person and that of the organism) and that ‘death’ is an ambiguous term which has one meaning when applied to people and a different meaning when used of other organisms; cf. Shrader (1986), McMahan (1995).

13 For further details about the data set, see Kitzinger and Kitzinger 2013, 2014.

14 One might question our extrapolating from comments by respondents, who are in extraordinary circumstances, to our ordinary concept of death. But the family member interviewees are ideal informants because they are not professionals working on neurological damage (medics, lawyers, etc.) yet they have been forced by circumstances to understand the condition and reflect on its ontological implications. They are non-naïve laypersons.

15 They also count heavily against sole reliance on the consciousness-based paradigm according to which PermVS patients are straightforwardly dead (see, e.g., Wikler 1988): our respondents are perfectly clear that it is apposite to talk about the patient, and not just their body, as being still alive.

16 An interesting variant on this is that some of our respondents spoke unguardedly of their relatives being dead before correcting themselves; e.g., ‘actually if Bella were alive, oh! say again, if Bella were awake, conscious and had got a mouthpiece, she would have …’

17 In contrast to Tim’s comments, Lizza (1993, p. 358–359) suggests that ‘when people understand the medical reality of [PermVS] they often engage in … ‘death behavior’’. But Lizza uses this to endorse his higher brain account on which PermVS patients are dead: ‘since no one finds such ‘death behaviour’ bizarre, it is socially acceptable. We thus have some reason to believe that society views … individuals in PermVS as dead’. By contrast, we argue that our respondents are in a quandary about their relative’s ontological status: their ‘death behaviour’ co-exists with repeated implicit and explicit statements that the patient is still alive, and with behaviour—such as Tim’s—which is concordant with the patient’s being alive.

18 The point was made in passing that no doubt our ordinary concept of death is even richer than the awkward conjunction of thoughts captured by the biological and consciousness-based paradigms. Other aspects of our understanding of death are provided by, for example, religious commitments and idiosyncratic beliefs. Evidence from the interview data supports this. For example, some interviewees struggled to explain where their relative’s soul currently resides. Tania asks rhetorically, “where’s his soul? No, his soul’s still- And I- I- I just thought that yeah, his soul has got to be there. Because I had this horror of it kind of like [pause] sort of floating around somewhere lo- and he was lost. You know like a lost soul […] Because he’s not- He’s still alive. He’s still breathing. His heart is still beating, so his soul is still obviously intact within his body.”

19
*Airedale NHS Trust v Bland* [1993]. Another neurologist reveals his uncertainty about the ontological status of PermVS patients whilst declining to accept the ethical distinction (though in practice of course implementing the legal distinction) between PermVS and WBD: “It’s only a legal nicety that defines brain stem dead people as dead at the time you make the diagnosis of ‘brain stem death’, rather than at the time they are dead. It's a legal nicety, that’s all. […] I think that brain stem dead people are as alive or as dead as somebody who is actually in the permanent vegetative state.”

20 This theme was present throughout the original Bland ruling: But for patients like Nancy Cruzan, who have no consciousness and no chance of recovery, there is a serious question as to whether the mere persistence of their bodies is ‘life,’ as that word is commonly understood… the idea of life is not conceived separately from the idea of a living person.
[…]To his parents and family he is ‘dead.’ His spirit has left him and all that remains is the shell of his body. This is kept functioning as a biological unit by the artificial process of feeding through a mechanically operated nasogastric tube. Intensive attention by skilled nurses assists the continuation of the existence of the body.
The fact that Anthony Bland’s existence will terminate does not in my judgment alter the reality that the true cause of death will be the massive injuries which he sustained in what has been described as the Hillsborough disaster. (All quotes from: [1993] 1 All ER 821 *Airedale NHS Trust v Bland*. http://ebookily.org/pdf/1993-1-all-er-821-airedale-nhs-trust-v-bland-124434330.html. Accessed 10 January 2014.)

21 Some previous research in which ordinary people reflect on the ontological status of people in PermVS supports our analysis. E.g., wives of patients in a persistent vegetative state refer to their husbands as “neither alive nor dead” (Hamama-Raz et al. 2013). Lotto et al. (2012) tested whether the perceived ontological status of PermVS, MCS, locked-in syndrome, and terminally ill patients, correlates with adherence to one of two life-ending principles, the sanctity of life principle (SL) and the principle of free choice (FC). They found that, ‘the more people believe in the FC, the more they perceived patients as dead in pathologies where conscious awareness is severely impaired. By contrast, participants who agree with the Sanctity of Life (SL) principle did not show differences across pathologies.’ This supports our view that people do not have a solely biological understanding of death: ‘for the supporters of the FC principle, as opposed to those who agree with the SL principle, conscious awareness seems to be central in defining what it does mean to be alive.’

22 Lotto et al. (2012) report a correlation between believing in the principle of free choice in life-ending decisions and perceiving *both* PermVS and MCS patients as dead. But this does not count against our claim that when people suggest that MSC patients are dead they are speaking metaphorically. Lotto et al.’s study consists of asking participants ‘how dead or how alive’ are certain sorts of patients, a methodology incapable of teasing out subtleties such as uncertainty about ‘death status’, and literal versus metaphorical uses of ontological terms. By contrast, our qualitative approach centring on in-depth interviews with relatives of PermVS patients is well suited to teasing out such subtleties. (For a similar critique of a different empirical study, see Holland 2010, p. 115.)

23 A terminological difficulty should be explicitly noted here. In our discussion we use phrases such as ‘life sustaining interventions’ and ‘bring about death’ for the sake of brevity: as is clear from the foregoing, our view is that the PermVS patient is not straightforwardly alive; but their currently unclear ontological state can be changed to one of being ‘really dead’ by withdrawing the support required to sustain their capacity for autonomic physiological functions. This terminological awkwardness is a general challenge for writers on this topic and sometimes results in new locutions, such as Miller and Truog’s (2010) preference for ‘somatic support’ as opposed to the clearly question-begging phrase, ‘life support’.

24 It is important to note that there are relatives of patients diagnosed as PermVS by clinicians who want the patient to be maintained in their current state. Thus far, in every case in our sample this is either because the family do not believe that the patient currently lacks, or do believe that the patient will recover, consciousness (and sometimes of course they are right).

25
*Re Quinlan, NJ 355* A 2d 647 (1976); *Cruzan v Director, Missouri Department of Health*, 497 US 261 (1990); *Bush v Schiavo,* 885 So 2d 321, 324 (Fla 2004); *Airedale NHS Trust v Bland* [1993].

26 These exchanges raise interesting issues we do not have space to pursue here, such as how to make sense of the fact that PermVS patients receive morphine despite being diagnosed as lacking conscious awareness, and how the doctrine of double effect applies to the ethics of ‘terminal sedation’ of PermVS patients.

27 The terminological difficulty explained in n. 23 is clearly prominent here: we use the term ‘euthanasia’ for convenience and with no implication that the PermVS patient is straightforwardly alive.

28 Others include the sanctity of life principle. The principle states that life is worthy of respect (although someone who adheres to the sanctity of life principle might acknowledge that there are times to allow death, e.g., by appealing to the doctrine of the double effect, or the distinction between proportionate and disproportionate interventions). The hidden premise in all versions of the principle, even secular ones, is that the individual in question is clearly alive; we suggest that application of the principle to PermVS patients is compromised by their unclear ontological status.

29 Opponents of euthanasia may continue to make the slippery slope objection to the suggestion that active euthanasia be allowed for PermVS patients, notwithstanding our argument. After all, psychological predispositions can be obdurate; and diagnostic uncertainties surrounding chronic disorders of consciousness encourage slippery slope worries (e.g., people with minimal consciousness may indeed have had CANH withdrawn because their state was seen as ‘very like’ a vegetative patient; see Huxtable 2013, p. 51ff). Nonetheless, the slippery slope argument against euthanasia is weakened and requires modifying in light of the finding that PermVS patients have a distinctive ontological status.

30 Although our argument does not rule out permitting active euthanasia for certain patients who are clearly alive, such as MCS patients, that discussion has yet to be had and, obviously, will not centre on the issue of ontological status. In passing we note that not even passive euthanasia has so far been permitted by the English courts for patients diagnosed as being in MCS (*Re M; W v M* (2011)).

31 Hence we acknowledge and accommodate evidence that some conscious and competent patients choose to refuse food and fluids in order to hasten their death, despite the availability of more active methods, such as physician assisted suicide (Ganzini et al. 2003).

## References

[CR1] *Airedale NHS Trust v Bland.* 1993. 2 WLR 316. http://www.bailii.org/uk/cases/UKHL/1992/5.html. Accessed 13 September 2013.

[CR2] Andrews K (1996). Misdiagnosis of the vegetative state: Retrospective study in a rehabilitation unit. British Medical Journal.

[CR3] Angell M (1994). After Quinlan: The dilemma of the persistent vegetative state. New England Journal of Medicine.

[CR4] Bernat J (1998). A defence of the whole-brain concept of death. Hastings Center Report.

[CR5] Bernat, J. 1999. Refinements in the definition and criterion of death. In *The definition of death: Contemporary controversies*, eds. S. J. Youngner, R. M. Arnold and R. Schapiro, 83–92. Baltimore, MD: Johns Hopkins University Press.

[CR6] Bernat J, Culver C, Bernard G (1981). On the definition and criterion of death. Annals of Internal Medicine.

[CR7] Borthwick C (1995). The proof of the vegetable: A commentary on medical futility. Journal of Medical Ethics.

[CR8] Borry P, Schotsmans P, Dierickx K (2004). Editorial: Empirical ethics: A challenge to bioethics. Medicine, Health Care and Philosophy.

[CR9] Brody B (1992). Special ethical issues in the management of PVS patients. Journal of Law, Medicine and Ethics.

[CR10] Constable C (2012). Withdrawal of artificial nutrition and hydration for patients in a permanent vegetative state. Bioethics.

[CR11] Cruse D (2011). Bedside detection of awareness in the vegetative state: A cohort study. Lancet.

[CR12] de Vries R, Gordijn B (2009). Empirical ethics and its alleged meta-ethical fallacies. Bioethics.

[CR13] Dyer C (2013). Researchers challenge findings of team that showed awareness in three patients in vegetative state. British Medical Journal.

[CR14] Fins JJ, Crowley Mary (2008). Brain injury: The vegetative and minimally conscious states. From birth to death and bench to clinic: The hastings center bioethics briefing book for journalists, policymakers, and campaigns.

[CR15] Fischer, D.B., and R.D. Truog. 2013. Conscientious of the conscious: Interactive capacity as a threshold marker for consciousness. *AJOB Neuroscience* 4(4): 26–33.

[CR16] Ganzini L (2003). Nurses’ experiences with hospice patients who refuse food and fluids to hasten death. The New England Journal of Medicine.

[CR17] Giacino JT (2002). The minimally conscious state: Definition and diagnostic criteria. Neurology.

[CR18] Goldfine AM (2013). Reanalysis of ‘Bedside detection of awareness in the vegetative state: A cohort study’. Lancet.

[CR19] Hamama-Raz Y, Zabari Y, Buchbiner E (2013). From hope to despair, and back: Being the wife of a patient in a persistent vegetative state. Qualitative Health Research.

[CR20] Hanser M (1999). Killing, letting die and preventing people from being saved. Utilitas.

[CR21] Holland S (2010). On the ordinary concept of death. Journal of Applied Philosophy.

[CR22] Huxtable R (2013). Law, ethics and compromise at the limits of life.

[CR23] Kitzinger, C., and J. Kitzinger. 2014. Withdrawing artificial nutrition and hydration from minimally conscious and vegetative patients: Family perspectives. *Journal of Medical Ethics*. doi:10.1136/medethics-2013-101799. 10.1136/medethics-2013-101799 PMC4316914 24425753

[CR46] Kitzinger, J., and C. Kitzinger. 2013. The ‘window of opportunity’ for death after severe brain injury: Family experiences. *Sociology of Health and Illness* 35(7): 1095–1112. 10.1111/1467-9566.12020 23278317

[CR24] Kopelman, L. M. 2007. Is withholding artificial nutrition and hydration from PVS patients active euthanasia? In *Pluralistic casuistry: Moral arguments, economic realities, and political theory (Philosophy and medicine, Vol 94*), eds. M. J. Cherry and A. Smith Iltis, 167–178. Netherlands: Springer.

[CR25] Lamb D (1996). Organ transplants and ethics.

[CR26] Laureys S (2010). Unresponsive wakefulness syndrome: A new name for the vegetative state or apallic syndrome. BMC Medicine.

[CR27] Lizza J (1993). Persons and death: What’s metaphysically wrong with our current statutory definition of death?. The Journal of Medicine and Philosophy.

[CR28] Lizza J (2006). Persons, humanity, and the definition of death.

[CR29] Lotto L (2012). Attitudes towards end-of-life decisions and the subjective concepts of consciousness: An empirical analysis. PLoS ONE.

[CR30] Marquet RL (2003). Twenty five years of requests for euthanasia and physician assisted suicide in Dutch general practice: Trend analysis. British Medical Journal.

[CR31] McMahan J (1995). The metaphysics of brain death. Bioethics.

[CR32] Miller FG, Truog RD (2010). Decapitation and the definition of death. Journal of Medical Ethics.

[CR33] Monti MM (2010). Willful modulation of brain activity in disorders of consciousness. New England Journal of Medicine.

[CR34] Multi-Society Task Force (1994). Medical aspects of the persistent vegetative state. New England Journal of Medicine.

[CR35] Nesbitt W (1995). Is killing no worse than letting die. Journal of Applied Philosophy.

[CR36] Rachels J (1975). Active and passive euthanasia. New England Journal of Medicine.

[CR37] *Re M: W v M *(2011) EWHC 2443 (COP). http://www.familylawweek.co.uk/site.aspx?i=ed86617. Accessed 13 September 2013.

[CR38] Rich B (1997). Postmodern personhood: A matter of consciousness. Bioethics.

[CR39] Royal College of Physicians. 2003. *The vegetative state. Guidance on diagnosis and management: Report of a working party of the Royal College of Physicians*. London: Royal College of Physicians. 10.7861/clinmedicine.3-3-249 PMC4952451 12848260

[CR47] Royal College of Physicians. 2013. *Prolonged disorders of consciousness: National clinical guideline: Report of a working party of the Royal College of Physicians*. London: Royal College of Physicians.

[CR41] Schnakers C (2009). Diagnostic accuracy of the vegetative and minimally conscious state: Clinical consensus versus standardized neurobehavioral assessment. BMC Neurology.

[CR42] Shrader D (1986). On dying more than one death. Hastings Center Report.

[CR43] Tooley M, Steinbock B (1980). An irrelevant consideration: Killing versus letting die. Killing and letting die.

[CR44] Veatch RM (1975). The whole-brain-oriented concept of death: An outmoded philosophical formulation. Journal of Thanatology.

[CR45] Wikler D (1988). Not dead, not dying? Ethical categories and persistent vegetative state. The Hastings Center Report.

